# High-Risk Neuroblastoma Treatment Review

**DOI:** 10.3390/children5090114

**Published:** 2018-08-28

**Authors:** Valeria Smith, Jennifer Foster

**Affiliations:** Baylor College of Medicine Department of Pediatrics, Texas Children’s Cancer and Hematology Center, Houston, TX 77030, USA; vs134101@bcm.edu

**Keywords:** neuroblastoma, high-risk neuroblastoma, diagnosis, treatment

## Abstract

Neuroblastoma is the most common extracranial solid tumor in children. One subset, high-risk neuroblastoma, is very difficult to treat and requires multi-modal therapy. Intensification of therapy has vastly improved survival rates, and research is focused on novel treatments to further improve survival rates. The current treatment schema is divided into three stages—induction, consolidation, and maintenance. This review serves as an overview of the current treatment for high-risk neuroblastoma and a glimpse at current research for future therapy.

## 1. Introduction

Neuroblastoma is the most common extracranial solid tumor in children, accounting for approximately 8% of all childhood cancers and 15% of childhood cancer mortality [1]. Patients with neuroblastoma are risk stratified based on a combination of radiographic, histologic, cytogenetic, and age at the time of diagnosis. Prior to the incorporation of intense multi-modal treatment, overall survival for patients with high-risk neuroblastoma (HRNBL) was less than 15% [1]. With the additions of radiation therapy, autologous stem cell transplant (ASCT), immunotherapy, and the differentiating agent isotretinoin, survival of HRBL now approaches 50%.

## 2. High-Risk Neuroblastoma Definition

The International Neuroblastoma Risk Group (INRG) utilizes a classification system that utilizes multiple risk factors to stratify patient’s pretreatment. These factors include INRG imaging stage, age, and pathology (histology, differentiation, amplification of MYCN, diploidy, and 11q aberration). The combination of these factors allows a patient to be stratified into the following pre-treatment risk groups: very low, low, intermediate, and high [2]. INRG stage (L1, L2, M, and MS) (Table 1) [3] classifies pre-treatment imaging on the basis of extent of diseases and image defining risk factors [4]. An age of 18 months (547 days) is the cutoff age distinction for most of this risk stratification, as those with a higher age at diagnosis typically have worse outcomes [2].

MYCN is an oncogene and it is considered amplified when more than four copies are present in the tumor; this is found in approximately 20% of neuroblastomas [5]. Patients with MYCN amplification are classified as high-risk, with the exception of the rare patient with L1 MYCN amplified disease that is completely resected. In addition, any patient with metastatic disease age 18 months or older is considered high risk irrespective of MYCN amplification.

## 3. Treatment

**Figure 1:**
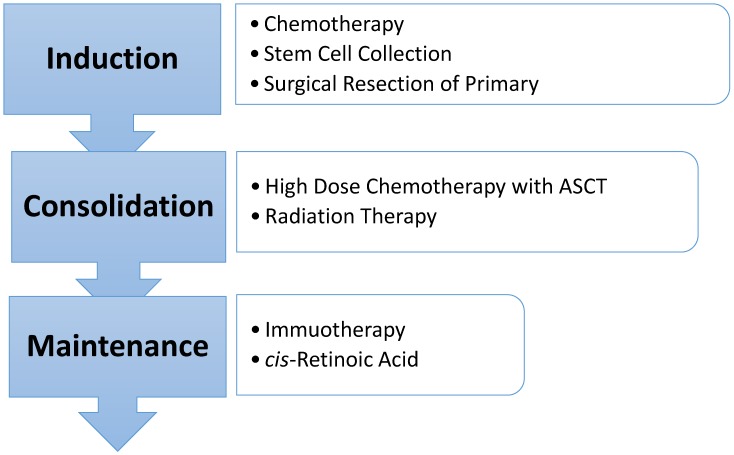
High-risk neuroblastoma treatment overview.

Current therapy in most institutions in North America is broken into three phases—induction, consolidation, and post-consolidation or maintenance therapy (Figure 1). Treatment includes chemotherapy, surgical resection, high-dose chemotherapy with autologous stem cell rescue, radiation therapy, immunotherapy, and isotretinoin. The current treatment lasts approximately 18 months.

There are slight variations on the induction regimens for high-risk neuroblastoma dependent upon where a patient is being treated. In general, during induction, patients receive 5–8 cycles of intensive chemotherapy including platinum, alkylating, and topoisomerase agents. Current North American induction regimens include vincristine, doxorubicin, cyclophosphamide, cisplatin, and etoposide. In addition, the Children’s Oncology Group (COG) trials have incorporated topotecan during the first two cycles of induction (NCT00004188, NCT00567567, NCT03126916). The Society of Pediatric Oncology Europe Neuroblastoma Group (SIOPEN) has utilized a rapid COJEC regimen that gives repeated cycles with compression of the recovery interval leading to fourteen-day cycles. Rapid COJEC gives eight total cycles utilizing combinations of vincristine, carboplatin, etoposide, cyclophosphamide, and cisplatin. A Cochrane review evaluating disease response and toxicities of rapid COJEC versus conventional induction therapy showed no difference in response and inconclusive data regarding toxicities [6]. A randomized trial of COJEC compared with the N5-MSKCC (Memorial Sloan Kettering Cancer Center) [7] regimen showed no differences in complete responses between the regimens but a lower frequency of toxicities with COJEC [8]. COJEC will thus remain the induction regimen for SIOPEN protocols.

During Induction patients undergo stem cell collection in preparation of their ASCT. Stem cells are collected either via periphery or bone marrow harvest. In COG protocols, stem cells were harvested post cycle 2 of induction and in rapid COJEC stem cells were harvested at the end of the eight induction cycles. As it is not uncommon for patients to have residual bone marrow disease at the time of stem cell collection, the COG conducted a study analyzing the impact of infusing autologous stem cell that had been purged of neuroblastoma cells versus infusing the stem cells without purging. The study showed no difference in survival (five-year event free survival (EFS) 40% vs. 36%, *p* = 0.77; five-year overall survival (OS) was 50% vs. 51%, *p* = 0.81) between purged vs. unpurged stem cells [9].

Surgery is another major component of HRNBL therapy and typically occurs at or near the end of induction chemotherapy. This timing is intended to maximize tumor shrinkage in an effort to minimize surgical morbidity. Data from a Children’s Cancer Group study showed that. in stage 4 tumors, there was not significantly improved survival for those with complete resection compared to those without complete resection, and the five-year EFS rate was 30% for patients who achieved complete resection compared with 25% (*p* = 0.1010) for those without. The ability to gain complete resection improved significantly, from 27% prior to chemotherapy to 45% with induction chemotherapy administration, supporting a delayed resection approach [10]. Similar results were reported from a German study, which showed that the extent of best operation had no impact on EFS [11]. In patients older than 18 months old with localized HRNBL, however, patients who underwent complete resection had superior local-progression-free survival (LPFS), EFS, and OS compared with patients who had gross total resections, incomplete surgery, or biopsy only [12]. Thus, in patients with metastatic high-risk disease, surgical morbidity should be carefully considered when planning resection and should not occur at the expense of a complete resection.

The consolidation phase follows induction with the goal of eliminating remaining minimal disease. Consolidation is divided into two parts and includes high dose chemotherapy followed by autologous stem cell transplant (ASCT) and radiation therapy. A landmark study evaluating the efficacy of stem cell transplant in treating patients with HRNBL showed significant improvement in EFS (three-year EFS 34% vs. 22% *p* = 0.034 and five-year EFS 30% vs. 19% *p* = 0.434) for those who underwent high dose chemotherapy with ASCT compared to those who completed continuation chemotherapy. There was, however, no difference OS (three-year OS 43% ASCT vs. 44% *p* = 0.87) [13,14]. A Cochrane review evaluating survival of patients who received autologous transplant versus continuation of chemotherapy versus no further treatment showed a statistically significant difference in EFS favoring the autologous stem cell transplant group but no significant difference in OS [15]. In order to further evaluate the role of ASCT in HRNBL, the COG conducted a randomized Phase 3 trial to compare tandem versus single ASCT (NCT00567567). In this study, patients were randomly assigned to receive single ASCT with CEM or tandem ASCT with thiotepa-cyclophosphamide followed by modified CEM (TC:CEM). The study concluded that patients who were randomized to TC:CEM had significantly higher EFS at three years compared with single ASCT (61.8% vs. 48.8% *p* = 0.0082) [16]. In patients who also received immunotherapy in maintenance, patients who were randomized to TC:CEM had significantly higher EFS and OS. A retrospective study compared tandem stem cell transplants to single ASCT and showed improved EFS (four-year EFS 59.3% vs. 26.8%, *p* = 0.01) without significant improvement in OS (four-year OS 70.6% vs. 44.2%, *p* = 0.06) for those receiving tandem transplants with similar results seen in COG [17].

Conditioning regimens for ASCT in patients with HRNBL have traditionally differed between North America and Europe. The COG had typically utilized CEM (carboplatin, etoposide, melphalan) while the Europeans utilized Bu/Mel (busulfan/melphalan). Studies to evaluate the two conditioning regimens have shown that Bu/Mel has less hepatic and renal toxicity compared to CEM [18]. A randomized Phase 3 trial showed improved EFS (five-year EFS was 45% vs. 33% *p* = 0.0005) and OS (five-year OS 54% vs. 41% *p* = 0.001) utilizing Bu/Mel compared to CEM with less overall toxicity [19]. The current COG trial ANBL1531 has patients assigned to Bu/Mel or modified CEM as a component of a tandem transplant (NCT03126916).

Radiation therapy typically occurs once the patient has recovered from ASCT and is associated with a high rate of local control. The standard amount of radiation administered is 21 Gy to the primary tumor bed, as well as radiation to end-induction sites of metastatic disease [20,21,22,23]. Current studies are ongoing, evaluating radiation dose escalation in patients with residual primary tumor and dose de-escalation in patients who had a complete resection of their primary tumor (NCT02245997). The few studies evaluating proton radiation therapy have shown that when compared to photon therapy, patients were able to decrease the dosage of radiation to nearby organs without any increase in local recurrence [24,25]. Though proton therapy is a potentially promising way to decrease toxicity of therapy, more research is needed given the small sample sizes in the existing publications. It is also important to note the potential issues of accessibility and cost when utilizing proton therapy in this patient population.

The post consolidation or maintenance phase of therapy was developed to treat residual disease that remains despite intensive induction and consolidation treatment regimens. In an effort to prevent the nearly 50% of patients who will relapse from doing so, the differentiating agent isotretinoin was added as an additional phase of therapy. In a randomized clinical trial, patients who received isotretinoin showed improvement in EFS, but not OS, over patients who did not [14,26].

In an effort to build on the success of incorporating treatment with isotretinoin to the end of consolidation therapy, immunotherapy with an anti-ganglioside 2 (GD2) was added to the isotretinoin backbone. Chimeric antibody 14.18 (ch14.18) is a monoclonal antibody against the tumor-associated diganglioside GD2. The COG completed a Phase 3 randomized trial evaluating post-consolidation patients receiving isotretinoin alone for six cycles or isotretinoin and ch14.18 in conjunction with the cytokines GM-CSF and IL-2. The patients randomized to the immunotherapy arm showed improved survival compared to isotretinoin alone (two-year EFS 66% vs. 46% *p* = 0.01, and two-year OS 86% vs. 75% *p* = 0.02) [27]. Other anti-GD2 antibodies have also shown a survival benefit [28]. Based on these results, standard of care includes maintenance therapy with isotretinoin and anti-GD2 therapy with cytokines. Maintenance therapy for HRNBL in Europe has a few differences than North America. In Europe, a different anti-GD2 antibody, dinutuximab beta, is used in conjunction with isotretinoin. Dinutuximab beta is combined with IL-2, and GM-CSF is not used in their maintenance regimens. Ongoing trials are evaluating the role of IL-2 administered subcutaneously as well as a long-term infusion of dinutuximab beta (NCT01704716).

## 4. Clinical Trials and Future Directions

Given the poor prognosis of high risk neuroblastoma, there are numerous studies evaluating treatments to improve survival. Several studies have evaluated and proven tolerability of MIBG in upfront therapy [29,30]. Given these results, the COG is conducting a Phase 3 study randomizing newly diagnosed patients with MIBG-avid disease to either standard induction or standard induction with the addition of I-131 MIBG therapy (NCT03126916). The molecular classification of neuroblastoma is another area of research interest in hopes of using this information for risk stratification or targeted treatments. The most frequent gene mutations found are in the following: ALK, PTPN11, ATRX, MYCN, and NRAS [31]. TERT mutations were found on whole genome sequencing in high risk neuroblastoma, although these only occurred in the absences of ATRX mutations or MYCN amplification [32]. Worse outcomes are seen in HRNB patients with distal 6q loss and amplifications not including MYCN [33]. Approximately 10% of spontaneous cases of high risk neuroblastoma have anaplastic lymphoma kinase (ALK) mutations and nearly all cases of familial neuroblastoma [5]. Crizotinib has been utilized in other cancers and shows promising results in preclinical patient derived tumor models when combined with induction chemotherapy drugs such as topotecan/cyclophosphamide [34,35]. A Phase 1 study of crizotinib in pediatric patients with refractory solid tumors or anaplastic large-cell lymphoma showed that the regimen was tolerable and warranted further investigation in neuroblastoma patients with ALK oncogenic mutations. The current COG led Phase 3 trial for patients with HRNBL has an Arm for patients with ALK mutations who will receive crizotinib in addition to standard therapy (NCT03126916). Immunotherapy with anti-GD2 antibodies have shown promising results in the upfront treatment of HRNBL. Current trials are incorporating this effect into novel delivery mechanisms during induction chemotherapy in an effort to maximize the immune response and increase therapeutic efficacy (NCT01857934).

The addition of a maintenance phase of therapy has improved survival. Studies are ongoing to determine if additional maintenance therapies can further prevent relapse and improve outcome. Difluoromethylornithine (DFMO) is an irreversible inhibitor of ornithine decarboxylase, an enzyme in polyamine synthesis first utilized as an anti-protozoan drug [36]. Based on results of a Phase 1 trial that showed DFMO to be tolerable for patients with relapsed or refractory neuroblastoma, a Phase 2 trial evaluating the effects of DFMO in patients in remission as part of an extended maintenance therapy is currently enrolling (NCT02139397) [37]. Another approach is the addition of a bivalent gangliosides (GD2 and GD3) vaccine in combination with β-Glucan for patients who are in remission. This regimen was proven tolerable in a Phase 1 study and is now being evaluated in a Phase 2 trial (NCT00911560) [38].

## 5. Conclusions

High-risk neuroblastoma requires intensive multimodality treatment to achieve the current survival rate of slightly less than 50%. Continued understanding of the biology of neuroblastoma will help to identify factors that change the outcomes of patients within this group, in particular identifying ultra high–risk neuroblastoma patients. Current research is focusing on further intensification of therapy to improve outcomes and evaluating the role of precision medicine in this patient population.

## Figures and Tables

**Table 1:** International neuroblastoma risk group (INRG) stages [3].

Stage	Description
L1	Localized tumor not involving vital structures as defined by the list of image-defined risk factors and confined to one body part
L2	Loco-regional tumor with presence of one more image-defined risk factors
M	Distant metastatic disease (except stage MS)
MS	Metastatic disease in children younger than 18 months with metastases confined to skin, liver, and/or bone marrow
